# Evaluating concentration estimation errors in ELISA microarray experiments

**DOI:** 10.1186/1471-2105-6-17

**Published:** 2005-01-26

**Authors:** Don Simone Daly, Amanda M White, Susan M Varnum, Kevin K Anderson, Richard C Zangar

**Affiliations:** 1 Statistical and Mathematical Sciences, Pacific Northwest National Laboratory, PO Box 999, Richland, WA, USA; 2 Biological Sciences, Pacific Northwest National Laboratory, PO Box 999, Richland, WA, USA

## Abstract

**Background:**

Enzyme-linked immunosorbent assay (ELISA) is a standard immunoassay to estimate a protein's concentration in a sample. Deploying ELISA in a microarray format permits simultaneous estimation of the concentrations of numerous proteins in a small sample. These estimates, however, are uncertain due to processing error and biological variability. Evaluating estimation error is critical to interpreting biological significance and improving the ELISA microarray process. Estimation error evaluation must be automated to realize a reliable high-throughput ELISA microarray system.

In this paper, we present a statistical method based on propagation of error to evaluate concentration estimation errors in the ELISA microarray process. Although propagation of error is central to this method and the focus of this paper, it is most effective only when comparable data are available. Therefore, we briefly discuss the roles of experimental design, data screening, normalization, and statistical diagnostics when evaluating ELISA microarray concentration estimation errors.

**Results:**

We use an ELISA microarray investigation of breast cancer biomarkers to illustrate the evaluation of concentration estimation errors. The illustration begins with a description of the design and resulting data, followed by a brief discussion of data screening and normalization. In our illustration, we fit a standard curve to the screened and normalized data, review the modeling diagnostics, and apply propagation of error.

We summarize the results with a simple, three-panel diagnostic visualization featuring a scatterplot of the standard data with logistic standard curve and 95% confidence intervals, an annotated histogram of sample measurements, and a plot of the 95% concentration coefficient of variation, or relative error, as a function of concentration.

**Conclusions:**

This statistical method should be of value in the rapid evaluation and quality control of high-throughput ELISA microarray analyses. Applying propagation of error to a variety of ELISA microarray concentration estimation models is straightforward. Displaying the results in the three-panel layout succinctly summarizes both the standard and sample data while providing an informative critique of applicability of the fitted model, the uncertainty in concentration estimates, and the quality of both the experiment and the ELISA microarray process.

## Background

Proteomic approaches are resulting in the identification of large numbers of proteins that can potentially be used as disease markers or drug targets. Unfortunately, proteomic approaches currently lack the throughput or quality metrics necessary to evaluate hundreds or thousands of samples that may be required to determine clinical usefulness of a biomarker [1]. Traditionally, candidate biomarkers have been commonly evaluated using a 96-well enzyme-linked immunosorbent assay (ELISA). However, this approach is not suited for analyzing more than a few proteins when sample volumes are limited, as is commonly the case for early tumor samples. For this reason, we and others are developing ELISA microarray systems to evaluate 20 to 50 proteins using only a few microliters of sample in an efficient and quantitative manner [2,3].

Processing a ELISA microarray experiment produces large volumes of data of wide variety and high complexity. Similar to traditional 96-well ELISA data, ELISA microarray data often are perturbed by processing error [4-7]. Processing errors are introduced by unintended variation in sample preparation, slide or pin arrangement, printing, imaging, and estimation of spot summary statistics. The specific role of concentration error estimates and the general role of statistical diagnostics is to reveal process accuracy and precision. This evaluation then enables an insightful interpretation of the biological significance, an informative critique of the current experiment, and insights to improve the accuracy and precision of future experiments. In a high-throughput ELISA microarray system, there is a need to not only quickly and accurately generate the standard curves and estimate concentrations from the sample data, but also to quickly evaluate the quality of those estimates. The resulting information can be used in both the development stage for optimizing assay conditions and in the production phase for ensuring that the overall analytic process is working well on a day-to-day basis.

Statistically evaluating ELISA microarray concentration estimation errors depends upon both the availability of the appropriate set of comparable measurements and the choice of data analysis methods. Sufficient replication within and across arrays is key to making precise estimates of both concentrations and errors [8]. Hence, evaluating concentration estimation errors in an ELISA microarray experiment begins with the design of the experiment. Evaluation of these estimation errors also depends on the recording of the pedigree, or history, of each result from probe preparation and array printing through sample preparation and spot intensity estimation [9-12]. Screening for anomalous results and normalizing within and across arrays may significantly reduce obscuring variation and improve homogeneity [13-15]. Although the mathematical statistics and algorithms are quite sophisticated, software makes actual estimation and application of the standard curve and the concentration error function straightforward. This is true also for the presentation of modeling results for diagnostic purposes.

In this paper, we describe and illustrate a methodology for calculating the concentration estimation error of each assay in an ELISA microarray experiment based on a statistical analysis of the most likely sources of error. We expect the resulting data analysis algorithms to be a key component in a bioinformatics package for evaluating ELISA microarray data.

## Methods

Making concentration estimates and estimating their errors in our ELISA microarray studies involve a sequence of steps beginning with the layout of the ELISA microarray and design of the experiment. Following execution of the analytical components of the experiment, the statistical analysis proceeds with data screening, normalization, and model identification. Estimation and evaluation of the standard curves and error estimation functions come next. Finally, the standard curves and error estimation functions are applied and then evaluated using a modeling diagnostic.

### Layout of the ELISA microarray and design of the experiment

To estimate errors in concentration estimates, it is necessary to carefully lay out the microarray and design the experiment. Our layout features several distally separate replicates of each assay spot on each microarray to evaluate local processing effects. Our design addresses selection and application of treatments – in particular, replicate treatments – to a collection of arrays. This replication facilitates adjustments for the sources of variability that lead to ambiguous concentration estimates [16,17]. In array experiments featuring relatively small numbers of assays, usually 50 or fewer analytes, thoughtful design is critical to normalization, calibration, and estimation of concentrations due to the significant lack of technical replicates found in arrays with thousands of assays. With regard to error estimation, the major consideration in the design of the experiments is replication of treatments across arrays to capture the effects of process error.

To illustrate our technique for evaluating estimation errors in an ELISA microarray experiment, we used a subset of data from an ELISA microarray investigation of breast cancer biomarkers. The ELISA microarray experiments were performed as previously described [2,3]. Briefly, capture antibodies were covalently attached to an aminosilanated glass slide surface (Sigma, St. Louis, Missouri, USA) using a Microgrid 2 robot from Genomic Solutions (Ann Arbor, Michigan, USA) equipped with ChipMaker2 split pins from TeleChem (Sunnyvale, California, USA). As demonstrated previously, these spots are typically uniform in shape with a reasonable homogenous distribution of protein across the spot [1-3]. That is, "donut" formation is not normally observed. These spatially confined antibodies bind a specific antigen from a sample overlaying the array. A second, biotinylated antibody that recognizes the same antigen as the first antibody but at a different epitope is then used for detection. Detection of the second antibody is based upon streptavidin (which binds biotin) and an enzymatic signal enhancement method known as tyramide signal amplification (TSA). The resultant fluorescence was detected at 10-micron scan resolution using a ScanArray 3000 from General Scanning (Billerica, Massachusetts, USA). The experiment used 94 arrays printed in pairs on 47 slides. Each array contained 4 (2 × 2) replicate subarrays of 25 (5 × 5) spots. A subarray contained 21 unique assays, 1 positive control and 3 negative control spots. A set of 7 known standard concentrations and a buffer blank was assembled by performing a three-fold dilution series of a single mixture of all the standards. Each standard concentration was applied to duplicate slides. The remaining 39 slides were treated with serum samples from women with or without breast cancer. These sera were encoded to prevent knowledge of the study group during sample processing. The treated microarrays were imaged with a ScanArray microarray scanner (PerkinElmer, Boston, Massachusetts, USA). The spot fluorescence estimates were calculated with custom array-image-analysis software that was developed in-house.

### Data screening, normalization and model identification

Data screening, an exploratory data analysis, serves several purposes – identifying outliers, anomalous values, and experimental design shortcomings; identifying data transforms to improve curve-fitting and application; identifying measurement trends and other processing effects; and suggesting an appropriate functional form for the standard curve [6,18-21]. This exploratory analysis combines simple summary statistics and graphical displays. For instance, graphs of control spot intensities versus processing variables such as array print order or pin number may reveal variability due to processing. These processing trends can be made more apparent with locally weighted regression, or loess, a statistical technique to fit a smooth curve through the scatterplot [22,23]. These graphs can be used as the basis for modifying the process or for data normalization.

Because our protein arrays feature fewer spots per array than do typical gene expression microarrays, a different approach to normalization, suitable for low spot-frequency arrays, is required. This normalization is critical, given that array-to-array processing error is common and that standard curves are estimated from reference spot intensities calculated from one set of arrays and then applied to sample spot intensities estimated from a separate set of arrays.

A scatterplot of intensity estimates of standard spots versus concentration is particularly useful. First, outliers and anomalies may be readily apparent. Second, the spacing between concentration values may be assessed. If standard concentrations follow from a dilution series, then the separation between concentrations decreases significantly with the decrease in concentration. This results in spot intensities measured at higher concentrations having much more leverage on the fit of the model than may be desirable. It should also be apparent whether the variability in spot intensity is increasing with mean spot intensity. Both increasing spacing in the concentrations and heteroskedasticity in the measured intensities affect the model fit and follow-on statistical inferences [24]. These may be minimized with log_*e *_transformations of both concentrations and spot intensities.

A scatterplot of raw or transformed standard spot intensity versus concentration also provides an indication of the appropriate model for the data. In particular, data following a sigmoid curve favor the logistic curves while data apparently lacking the horizontal asymptotes of a sigmoid curve favor a linear or power law model. Although several models may be fit and one selected based on a goodness-of-fit statistic (see next section), the scatterplot is a useful visual check on this selection.

Several plots provide useful information about the quality of the fitted model. Of special importance are the scatterplot of residuals versus concentration and the scatterplot of residuals versus estimated intensity. In both cases, the variability of the residuals should be centered about zero and constant across concentration or intensity. Model bias is indicated by a systematic drift of residuals to one side of the zero line. Heteroskedasticity is indicated by a systematic change in the variation of the residuals. Both may indicate that a better model is necessary before proceeding to estimation of sample concentrations and estimation of concentration errors.

### Standard curves and estimation errors

An ELISA standard curve expresses protein concentration as a function of spot intensity. One standard curve is required for each assay. In an ELISA microarray experiment, the standard data are collected by fixing a set of concentrations and measuring spot intensities via imagery of the treated arrays. A standard curve is estimated by fitting an appropriate function to the set of (concentration, intensity) measurement pairs [25]. This equation is then inverted to obtain the standard curve.

Common parametric choices for standard curve models are multiparameter logistic functions and power law functions. For an ELISA microarray, a strictly monotone model is consistent with the belief that a monotone change in concentration should result in a monotone change in spot intensity.

We estimate standard curves with both logistic and power law parametric models. The four-parameter logistic model [26], expressing intensity *I *as a function of concentration *C *and parameters *P*_1_, *P*_2_, *P*_3 _and *P*_4_, is defined as


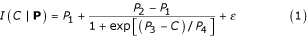


The two-parameter power law model [27] expressing intensity *I *as a function of concentration *C *and parameters *P*_1 _and *P*_2_, in log_*e *_terms, is

log_*e *_(*I*) = *P*_1 _+ *P*_2 _log_*e *_(*C*) + *ε*

We assume the errors, denoted by the term *ε*, are independent and normally distributed with mean 0 and variance *σ*^2^. With either of these parametric models, concentration estimation errors may be estimated using propagation of error, also known as the delta method.

To choose between competing candidate models, a number of measures exist for evaluating model fit when replicate observations of each assay are available. These include partitioning the mean squared error, or MSE, into components representing pure error and lack of fit [28], and penalized measures such as Akaike (AIC) and Bayesian (BIC) information criteria [29]. We also examine the *PRESS *statistic, a direct measure of the predictive capability of each candidate model [30].

To calculate the *PRESS *statistic for each candidate model, suppose we exclude each poin (*x*_*j*_, *y*_*j*_) in turn and fit the model to the remaining points. We predict the value 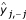 at the excluded point *x*_*j *_and calculate the *PRESS *residual defined by *e*_*j*, - *j *_= *y*_*j *_- 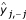. Then, the *PRESS *statistic is the sum of the squared *PRESS *residuals


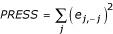


The candidate model with the lowest *PRESS *score as the best predictive model to estimate concentrations.

The basic approach to estimating concentration errors with the propagation of error method has three steps [31]. First, fit intensity as a function of concentration and estimate the covariance among model parameter estimates. Next, solve the fitted function for concentration as a function of intensity. Finally, propagate error estimates from the fitted model through the inverted model and combine with the error estimate of the sampled spot intensity to estimate the concentration estimation error.

Let *C*(*I*|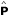), with 
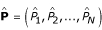
, denote the inverted *N *parameter model expressing concentration *C *as a function of intensity *I *and the parameter estimates 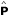 Suppose 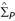 is the *NxN *parameter covariance matrix estimated by fitting *I *as a function of *C*, say *I*(*C*|**P**). Now, let *C*_*s *_be the estimated concentration from the sample intensity estimate *I*_*S*_, say *C*_*S *_= *C*(*I*_*S*_/**P**) and 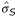 be the corresponding estimated standard error of *I*_*S*_. Then, the propagation of error estimate for the concentration estimate *C*_*S *_is the square root of the product of 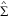, the sample covariance matrix augmented with 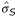, and the Jacobian matrix *J *evaluated at *I*_*S *_and the parameter estimates 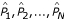. In this application, the Jacobian is the matrix of partial derivatives of *C*(*I*|**P**) with respect to the intensity *I *and the parameters **P**. Hence, the concentration estimation error of *C*(*I*|**P**) is the square root of the concentration estimation variance *V*(*C*(*I*|**P**))

*V*(*C*(*I*|**P**)) = *J*(*C*(*I*|**P**))^*T *^Σ*J*(*C*(*I*|**P**))

where the Jacobian is

*J*(*C*(*I*|**P**))^*T *^= [∂*C*/∂*I*, ∂*C*/∂*P*_1_,..., ∂*C*/∂*P*_*N*_]

and the augmented covariance matrix is


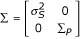


Hence, the formula for estimated standard error of *C*_*S *_is


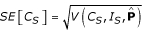


For a given intensity estimate *I*_*S *_and standard error 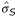, the estimated concentration and approximate 95% confidence interval (*C*_95%*L*_, *C*_95%*U*_) are

*C*_*S *_= *C*(*I*_*S*_)

*C*_95%*L *_= *C*_*S *_- 2*SE*[*C*_*S*_]     (2)

and

*C*_95%*U *_= *C*_*S *_+ 2*SE*[*C*_*S*_]     (3)

For example, consider the four parameter logistic model, Eqn. 1. The concentration estimation equation is obtained by solving this equation for *C *in terms of *I *and the four parameters


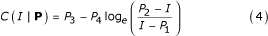


The Jacobian matrix is obtained by taking the partial derivatives of the inverted four-parameter logistic function of *C *(Eqn. 4) with respect to *I *and the parameters *P*_1_, *P*_2_, *P*_3 _and *P*_4_


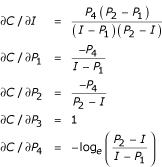


### Diagnostic visualizations

A three-panel display combining a histogram of normalized sample spot intensities for a given antigen, its corresponding standard curve, and the graph of the concentration coefficient of variation, or relative error, versus concentration provides pertinent information about the conduct of the current experiment as well as information to improve future experiments. The standard curve panel presents a scatterplot of normalized standard spot intensities versus standard concentrations. The scatterplot is overlain with the estimated standard curve expressing concentration as a function of spot intensity. This panel also includes approximate 95% confidence intervals. These intervals summarize the uncertainty in concentration estimates due to both the uncertainty in estimating the standard curve and the uncertainty in the sample spot intensity estimate. Finally, a highlighted region helps distinguish concentration estimates s with acceptable errors from concentration estimates with possibly less than acceptable errors.

The segment of the standard curve corresponding to acceptable concentration errors may be determined using the 95% confidence intervals. The lower and upper endpoints of this segment, (*I*_*L*_, *C*_*L*_) and (*I*_*U*_, *C*_*U*_), are the two points such that the confidence intervals begin to increase significantly in length. This segment generally corresponds to the linear segment of a standard curve. We identify the intensity *I*_*L *_of the lower pair as the smallest intensity such that 95% UB(*I*_*L*_) is less than 95% UB(*I*) for intensity values *I *less than *I*_*L*_. Similarly, we identify *I*_*U *_as the largest intensity such that 95% LB(*I*_*U*_) is greater than 95% LB(*I*) for intensity values *I *greater than *I*_*U*_. We define *C*_*L *_and *C*_*U *_to be *C*_*L *_= *C*(*I*_*L*_) and *C*_*U *_= *C*(*I*_*U*_), respectively. We believe that this is a conservative approach to identifying intensities that generate concentration estimates with acceptable errors.

An informative visualization of acceptable concentration estimates may be generated using the points (*I*_*L*_, *C*_*L*_) and (*I*_*U*_, *C*_*U*_). Consider the union of the two rectangular regions defined by the two sets of vertices [(*I*_*L*_, 0), (*I*_*L*_, *C*_*L*_), (*I*_*U*_, *C*_*U*_), (*I*_*U*_, 0)], and [(0, *C*_*L*_), (0, *C*_*U*_), (*I*_*U*_, *C*_*U*_), (*I*_*U*_, *C*_*LU*_)]. This union defines an L-shaped region covering the standard curve segment and bound at its extremes by the intensity and concentration segments. From this visualization, one can quickly grasp the dynamic range of acceptable intensities and the potential range of acceptable concentration estimates.

In regard to this first panel, two notable aspects of this propagation of error methodology are noteworthy. First, the error bands are computed pointwise and provide reasonable error estimates for a small number of concentrations. As the number of concentration estimates grows, the impact of the multiple testing problem grows [32]. This a problem in any biomedical testing that features numerous simultaneous tests and has spawned considerable debate and research. The second aspect of note is the divergence of the error bands from the estimated standard curve as the standard curve approaches a horizontal asymptote. We see this apparent deficiency in the method as a plus. This divergence is a clear indicator that concentration estimates in the segment of a standard curve approaching a horizontal asymptote are highly suspect.

The second panel in this display shows the concentration coefficient of variation – that is, %*CCV *= 100 * *SE*(*C*|*I*)/*C*(*I*), or relative error of a concentration estimate – as a function of concentration. This provides an alternative view of the error in concentration estimation over the concentration range covered by the concentration estimation equation. A standard curve modeled with a four-parameter logistic function generally will have a bathtub shape due to the increasing uncertainty in concentration estimates at the two ends of the concentration range where the curve approaches horizontal asymptotes.

The third panel in this display features an annotated histogram of sample spot intensity estimates on the intensity axis opposite the scatterplot. In this representation, it is easy to see the extent of overlap between the distribution of sample intensity estimates and the range of intensities that result in concentration s estimates with acceptable errors.

## Results and discussion

**Figure 1:**
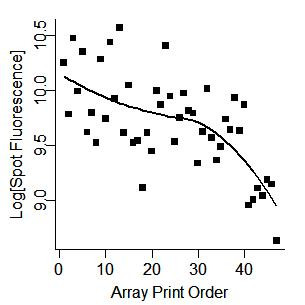
**Control spot fluorescence and slide print order **We expect control spot intensity to be constant across slide print order. The trend, estimated with loess regression, indicates a processing effect due to printing order. In this case, the decrease in control spot intensity, coupled with knowledge of the printing process, suggests that the quantity of printed material is decreasing over a printing run.

To evaluate concentration estimation errors in the example analysis, we attempted to quantify or understand those errors that we can and minimize those errors that we cannot. We began with data screening. The most significant anomaly uncovered during this exploratory analysis of the cancer biomarker data was a decreasing trend in control spot intensity as a function of array print order (Figure 1). The trend was quantified using loess, a flexible, nonparametric method to fit a smooth curve through a scatterplot to uncover trends in data [22,23]. This trend suggests that 1) normalizing across arrays would improve precision; 2) in future experiments, assigning study groups to arrays should address array print order; and 3) array printing should be monitored and, if possible, modified to reduce this source of obscuring variation. In this case, we normalized for slide-level processing errors by subtracting from each spot's log_*e*_(fluorescent intensity) the difference between the mean of its slide's control spot log_*e *_(intensities) and the corresponding loess estimate.

**Figure 2:**
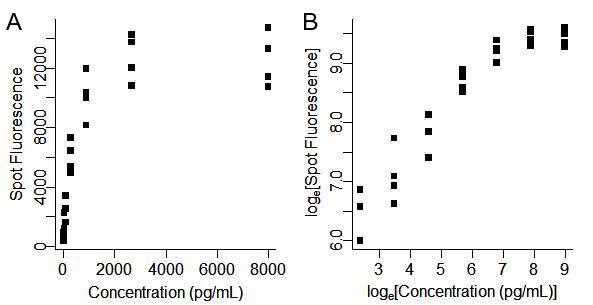
**Spot fluorescence and standard concentration **A graph of spot fluorescence as a function of standard concentration (A) shows that both the difference in concentration and the variability in spot fluorescence increase with concentration. Both negatively affect model fitting and subsequent statistical inferences due to the excessive leverage of the larger concentrations and the heteroskedasticity of the spot intensities. In this case, log_*e *_transforms of both concentration and spot fluorescence reduce the excessive leverage and improve the homogeneity (B). The log transformation is consistent with the featured concentration dilution series and is a common transformation of chemical measurements that often are assumed log normally distributed.

Our evaluation addressed the selection and fitting of an acceptable concentration estimation model. To that end, we examined two plots. The first displays the fluorescent intensities of the standards as a function of concentration (Figure 2A). Two characteristics of the data that significantly affect selecting and fitting the model and then interpreting the results in a statistically meaningful way are apparent.

The first is heteroskedasticity, or the increasing variation in fluorescent intensity with increasing concentration. Meaningful statistical inferences about concentration estimation errors depend upon correct modeling assumptions. To apply propagation of error when estimating and then interpreting the approximate 95% confidence intervals, we rely on normal distribution theory and require that the random variability in spot intensities be homogeneous across concentrations [28]. In this case, a log_*e *_transformation of the intensity estimates stabilizes the variability across concentrations (Figure 2B).

The second characteristic is the undue leverage of data at high concentrations due to the increasing separation between standard concentrations with increasing concentration. Although both are expected (the first due to the randomness generally observed when counting photons, and the second due to the use of a concentration dilution series in the design), each must be addressed to achieve the best fit of the standard curve and resulting concentration estimation inferences. A log_*e *_transformation of the concentrations standardizes the separation in concentrations (Figure 2B).

**Figure 3:**
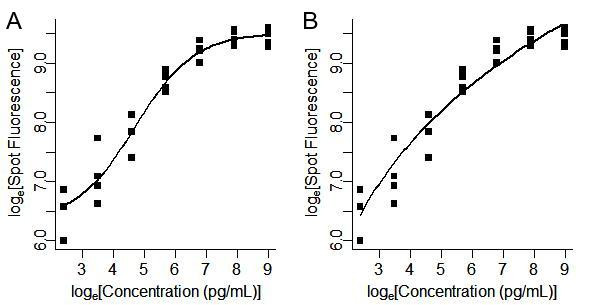
**Candidate standard curves **A review of the graphs of a four-parameter logistic curve (A) and a power law curve (B) suggests that the former shows higher fidelity to the data and is a better choice for the standard curve in this case.

**Figure 4:**
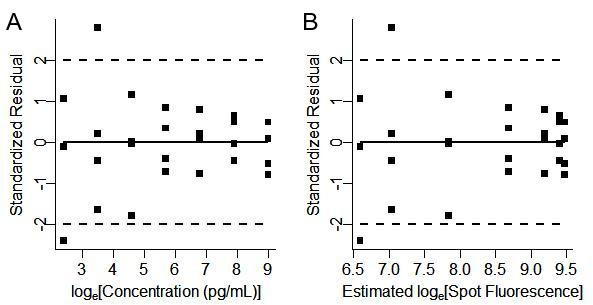
**Modeling diagnostics **The points in graphs of the standardized residuals versus the log_*e *_standard concentrations (A) and the standardized residuals versus the estimated log_*e *_spot intensities (B) are reasonably well behaved, showing no strong systematic trends or deviations from the zero line. These indicate that the four-parameter logistic model is acceptable, that subsequent statistical inferences are reasonable, but that a better model may exist in another model family.

With the heteroskedasticity and undue leverage addressed, we estimated a standard curve by selecting one of two models: a four-parameter logistic model (Eqn. 1) and a power curve model (Eqn. ??). We chose the logistic model as the model that fits the data best visually and in terms of the PRESS statistic (Figure 3). The logistic curve more closely follows the data points, while the power curve is too high in the lower concentrations and too low in the higher concentrations. We confirmed our choice with a review of the modeling diagnostics. In this case, we examined graphs of the standardized residuals as a function of concentration and the estimated intensities (Figure 4). In both graphs, the residuals show no significant systematic trends or deviations from the zero line and vary uniformly. Further, the preponderance of standardized residuals falls between -2 and 2, indicating that a statistical interpretation of the 95% confidence intervals is warranted.

**Figure 5:**
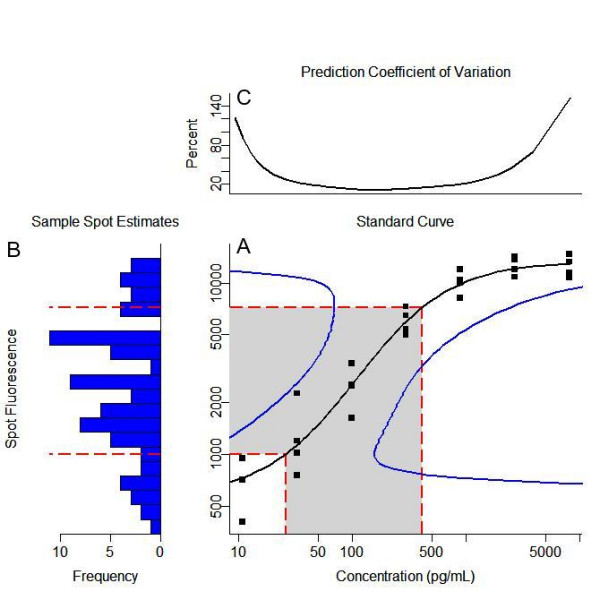
**Three-panel diagnostic summary **The standard curve panel (A) of the three-panel diagnostic summary features a scatterplot of the data, the estimated standard curve (black line), the 95% confidence intervals (blue lines), and the region of acceptable errors (grey). For this example, the acceptable segment of the standard curve (i.e., the segment with concentration estimation errors acceptable to us) covers spot intensities from about 1000 to 7500 intensity units and concentrations from about 25 to 500 pg/ml. The histogram of sample intensities (B) suggests that about one-fifth of the sample spot intensities is below the detection limit of about 1000 units, about three-fifths will produce estimated concentrations in the acceptable range, and one-fifth exceeds the acceptable range. With regard to remeasuring the standards in this experiment or imaging in future experiments, the three-panel graph suggests that it may be worthwhile to attempt to extend the acceptable range. It also suggests that the normalization between the standards measurements and sample measurements should be revisited. The graph of the concentration coefficient of variation as function of concentration (C) offers an alternative summary of the estimation errors over the range of concentrations.

Figure 5 presents the three-panel diagnostic visualization for the HER-2 data. HER-2 belongs to the family of epidermal growth factor receptors and has been used as a serum biomarker for the detection of breast cancer. This figure illustrates how data from a large study measuring HER-2 levels in the serum of women with and without breast cancer can be visualized using this statistical approach. A standard curve of HER-2 was generated, and the concentration of HER-2 in 39 samples was determined. To estimate manually the concentration for a sample HER-2 spot intensity, say I, locate I on the vertical axis, then scan across horizontally to the standard curve and 95% confidence intervals (Figure 5A). Scan down from these points to find the appropriate estimated concentration and lower and upper 95% concentration confidence bounds. In this manner, the estimated concentration and confidence interval can be determined.

In the standard curve panel (Figure 5A), we see that near the asymptotes of the standard curve, the uncertainty grows much more quickly than the curve, causing the concentration confidence bounds to diverge. Although this divergence is due to the approximation (Eqns 2 and 3), it is true that near the asymptotes, the uncertainty of the estimated concentration increases greatly. For this reason we have defined our optimal region of this curve to be the range of spot fluorescence values such that both the upper and lower bounds are monotonically increasing in intensity. The boundaries of this range are indicated by the shaded area and the dashed red lines, which also show the concentration values corresponding to the acceptable fluorescence range. Our optimal concentration range spans approximately two orders of magnitude.

The histogram (Figure 5B) shows the fluorescence values of the sample spots for which this curve may best be used to estimate concentration. The plot shows that many of the sample values lie outside our optimal region. The researcher must then decide if too many of these values lie outside this range and, if so, what can be done to fix this problem. Nevertheless, we were able to compare the HER-2 concentrations and found a 3.5-fold increase in HER-2 protein levels in women with stage Ill/stage IV breast cancer (7 samples) compared to women without breast cancer (12 samples).

The concentration coefficient of variation, or the ratio of the concentration estimation error to the corresponding estimated concentration, offers an alternative expression to a confidence interval as a means to evaluate concentration estimation error. A graph of this estimation error as a function of concentration offers a comprehensive summary of the variation in the concentration coefficient of variation over the concentration range (Figure 5C).

Presenting the results in this type of plot allows us to immediately look for several potential problems. First, does the fitted curve seem reasonable, given the data points to which we are fitting? We also can determine whether most of the unknown sample data fall within the acceptable range of the curve. The usable concentration range is made clear and, if it is too limited in range, it is immediately apparent. If problems are identified, several fixes are available, including changing the settings on the imager or using a different concentration range to create the standard curves.

## Conclusions

Evaluation of errors in estimating concentrations is important to establishing confidence in protein concentration estimates. Propagation of error provides a straightforward approach to estimating concentration estimation errors in ELISA microarray experiments. When presented in a simple multi-panel visualization, the propagated errors provide valuable information about individual concentration estimates, the applicability of the estimated standard curve, quality of the experiment, and the conduct of the ELISA microarray processing. The visualization provides a rapid assessment of the quality of the data, particularly in regard to the goodness of fit of the estimated standard curve and its capability to estimate concentrations over the observed range of intensities of biological samples.

## Authors' contributions

DSD framed evaluation of ELISA microarray concentration estimation errors as a statistical problem. KKA suggested the use of propagation of error and outlined the initial derivation. DSD and AMW derived the appropriate propagation of error equations, developed the algorithms, and designed the diagnostic visualizations. AMW encoded the algorithms, analyzed the microarray imagery, and produced the statistical results. SMV designed and printed the arrays and then carried out the ELISA microarray experiments. RCZ conceived the study, designed it with SMV, and then coordinated all our efforts. All authors submitted comments on drafts, then read and approved the final manuscript.
